# The Effect of Mirid Density on Volatile-Mediated Foraging Behaviour of *Apolygus lucorum* and *Peristenus spretus*

**DOI:** 10.3390/insects12100870

**Published:** 2021-09-25

**Authors:** Han Chen, Honghua Su, Shuai Zhang, Tianxing Jing, Zhe Liu, Yizhong Yang

**Affiliations:** College of Horticulture and Plant Protection, Yangzhou University, Yangzhou 225007, China; levyashin276@163.com (H.C.); susugj@126.com (H.S.); shuaizhang@yzu.edu.cn (S.Z.); jingtx@yzu.edu.cn (T.J.); zhe_liu9208@163.com (Z.L.)

**Keywords:** behavior, pest, natural enemy, HIPVs, density

## Abstract

**Simple Summary:**

Since the widespread adoption of Bt cotton in the late 1990s, the green mirid bug, *Apolygus lucorum* (Hemiptera: Miridae), has become one of the most important pests in cotton fields and some other crops. To manage this destructive pest, *Peristenus spretus* (Hymenoptera: Braconidae) has been tested in augmentative biological control. In this study, after cotton plants were damaged by different densities of *A. lucorum*, the behavioral responses of *A. lucorum* and *P. spretus* to cotton plants volatiles were evaluated, and the quality and quantity of volatiles from cotton plants were analyzed. The results demonstrated that HIPVs emitted by plants in response to *A. lucorum* could be influenced by the pest density and could be identified by *P. spretus* as a signal of the host. Our results would help understand how *P. spretus* plays a role in biological control against *A. lucorum*.

**Abstract:**

Plants would release herbivore-induced plant volatiles (HIPVs) to repel herbivores and attract natural enemies after being damaged by herbivores. In this study, after cotton plants were damaged by different densities of *Apolygus lucorum*, the behavioral responses of *A. lucorum* and *Peristenus spretus* to cotton plants volatiles were evaluated, and the quality and quantity of volatiles from cotton plants were analyzed. Only when cotton plants were damaged by four bugs did both *A. lucorum* and *P. spretus* show an obvious response to damaged cotton plants, which indicates that cotton defense is correlated with pest density. The collection and analysis of volatiles reveals that the increase in pest density results in the emission of new compounds and an increase in the total number of volatiles with an alteration in proportions among the compounds in the blend. These changes in volatile profiles might provide wasps and mirids with specific information on host habitat quality and thus could explain the behavioral responses of parasitoids and pests.

## 1. Introduction

Many herbivores attacked plants during their growth; however, plants were not merely passive victims of herbivore attacks, and a variety of mechanisms that contributed to their resistance would be reconfigured to tolerate the damage and stresses of natural ecosystems [1,2,3,4,5]. Different chemical defense responses were induced by herbivory arthropods in different plants [6]. The important life-history parameters of herbivory arthropods such as development time, fecundity, longevity, or survival were negatively affected by some non-volatile toxic secondary metabolites. Meanwhile, subsequent repellency of herbivores and attraction of natural enemies were both determined by the herbivore-induced plant volatiles (HIPVs) [7,8,9,10].

HIPVs were always composed of two classes [11]. The first class was called constitutive volatile compounds, which mainly comprised green leaf volatiles (six-carbon alcohols, aldehydes, and acetates) emitting immediately from ruptured plant cells after mechanical damage [12,13,14]. The second class was called inducible volatile compounds, which have always been produced as a result of insect damage or oviposition [15,16]. They were emitted from the site of the wound and lasted for a period after herbivore removal [17,18].

HIPVs always play crucial roles in tritrophic interactions between plants, herbivores, and carnivores, for the timely emission of specific HIPVs determined the effectiveness of volatile-mediated defense response. Previous studies have demonstrated that HIPVs affect behaviors of herbivorous or carnivorous arthropods [7,19,20]. However, although different HIPVs might share many compounds, the amount and composition of HIPVs were still different among plant species [21]. In addition, the amount and constitution of HIPVs were also influenced by pest density and were specific to each plant-pest-natural enemy association [22,23].

A lot of prior efforts have shown that herbivore density influences the intensity of feeding damage in to modulate interactions between plants and insects; meanwhile, the attractiveness of herbivore-infested plants to parasitoids and predators were also changed [8,14]. However, the knowledge about how different herbivore densities caused dynamic changes in HIPVs composition and, thus, how to change the behaviors of pests and natural enemies were still limited.

Since the widespread adoption of transgenic *Bacillus thuringiensis* (Bt) cotton in the late 1990s, the green mirid bug, *Apolygus lucorum* Meyer-Dür (Hemiptera: Miridae), has become one of the most important pests in cotton fields and some other crops [24]. To manage this destructive pest, the native endoparasitoid *Peristenus spretus* Chen et van Achterberg (Hymenoptera: Braconidae) has been used in augmentative biological control. A series of studies confirmed that *P. spretus* was attracted by *A. lucorum* damaged cotton plant, and the parasitism rate was about 30%. This indicated that *P. spretus* had great potential in biological control against *A. lucorum* [25].

In order to better understand the impact of pest density changes on HIPVs and the response of *A. lucorum* and *P. spretus* to cotton plants infestation by *A. lucorum*, the HIPVs from cotton plants damaged by *A. lucorum* under different densities were collected. Our objectives were (1) to investigate whether the behavior of *A. lucorum* and *P. spretus* varied substantially with herbivore densities and (2) to analyze the component complexity in HIPVs from different densities.

## 2. Materials and Methods

### 2.1. Insects and Cotton Plants

For the experiment, the colonies of *A. lucorum* and *P. spretus* were established. Both colonies were provided by the laboratory at the Langfang Experiment Station, Chinese Academy of Agricultural Sciences (CAAS), Hebei Province, China (39.53° N, 116.70° E). The *A. lucorum* was reared on fresh green bean (*Phaselus vulgaris* L.) pod and maize (*Zea mays* L.) in the laboratory of Yangzhou University at 25 ± 1 °C with 60 ± 10% RH and a 14:10 (L:D) photoperiod (the light period starts at 5:00 a.m.) in a climate-controlled rearing chambers [26]. The *P. spretus* was also reared in the growth chamber of Yangzhou University at 25 ± 1 °C with 65 ± 5% RH and a 14 h light:10 h dark (the light period starts at 5:00 a.m.). They were reared on the second instar nymphs of *A. lucorum* and maintained with a 10% honey solution [27].

The seeds of transgenic Bt cotton (var. SGK321) were provided by the Biotechnology Research Institute of CAAS (Beijing) and grown in the greenhouse of Yangzhou University. All the plants were enclosed in a nylon cage with 60 mesh to prevent herbivore damage. After 8 leaves came out, similar and healthy plants were collected for experiments.

### 2.2. Cotton Plants Treatment

The cotton plants were individually placed in the collection apparatuses for 1 day before volatile collection to adapt to the new environmental condition. Before treatment, 3rd-stage-old nymphal *A. lucorum* specimens were starved for 8 h and then carefully transferred to true leaves of cotton by a soft brush. The following treatments were used: control undamaged plant (CK) and each plant infested with 1 miridae (1 bug), 2 miridae (2 bugs), 4 miridae (4 bugs) and 8 miridae (8 bugs). The experiment started at 10:00 a.m., and after 24 h of initial *A. lucorum* infestation, the nymphals were removed. All densities treatments were replicated four times.

### 2.3. Behavioral Assays

Y-tube olfactometer bioassays were conducted to evaluate the behavior responses of *A. lucorum* and *P. spretus* to cotton plants’ volatiles. The equipment was similar to that of Xiu with slightly modified procedures [20]. Briefly, a glass jar (capacity: 10 L) was connected to each of the two arms of the Y-tube olfactometer. Each glass contained an air inlet tube connecting a vacuum pump (QC-1, Beijing Institute of Labor Instrument, Beijing, China) and an outlet tube connecting to the olfactometer arm. The vacuum pump pushed air through activated charcoal and an Erlenmeyer flask filled with distilled water. Airflow through each arm was maintained at 1 L/min. Different treatments of cotton plants were placed inside the glass jar for experiments.

All the *A. lucorum*-induced cotton plants volatiles (1, 2, 4, and 8 bugs) were tested against control volatiles (healthy). *Apolygus lucorum* or *P. spretus* was released individually at the entrance of the Y-tube olfactometer and was allowed only 5 min to respond to the two odor sources. The number of insects choosing each of the odors was recorded. Sixty females of *P. spretus* were tested from 10:00 a.m. to 14:00 p.m., while sixty adults of *A. lucorum* were tested from 17:00 p.m. to 21:00 p.m. because the feeding and parasitic behaviors happened during these two periods in the field, respectively [26,27]. All parasitoids and pests used in the experiments were 2–4 days old.

### 2.4. Headspace Volatile Collection and Analysis

The collection apparatus for volatile emitted by healthy and damaged cotton plants involved a dynamic headspace collection system [28]. Briefly, the plant’s pot was removed, and the roots with soil were packed in aluminum foil. After that, the plant was transferred to a 10-L glass jar. Before trapping volatile, the system was purged at a flow rate of 1 L/min with a vacuum pump (QC-1, Beijing Institute of Labor Instrument, Beijing, China) for more than 30 min. During the volatile collection, activated charcoal purified air was pumped through Teflon tubing into the system at a flow rate of 1 L/min through a glass tube (length: 89 mm, inner diameter: 5 mm) filled with 100 mg Tenax TA (60/80 mesh; Sigma-Aldrich, Oakville, ON, Canada).

The headspace volatiles from plants was collected for a 4 h period from 10:00 a.m. to 14:00 p.m. and 17:00 p.m. to 21:00 p.m., respectively. After collection, the volatiles were extracted with 400 μL n-hexane (Sinopharm Chemical Reagent Co. Ltd., Shanghai, China) and 4 μg n-octane (Aladdin Co. Ltd., Shanghai, China) was added to each sample as an internal standard.

The volatile samples were then analyzed using a coupled gas chromatography-mass spectrometry (GC-MS) instrument (Trace ISQ, Thermofisher, CA, USA) equipped with a DB-5 MS column (30 m × 0.25 mm × 0.25 μm). Sample injection was operated splitlessly at 200 °C, and helium was used as the carrier gas with a flow rate of 1 mL/min. The column temperature was maintained at 40 °C for 1 min and then programmed from 5 °C/min to 210 °C, and finally from 15 °C/min to 250 °C. The ionization was by electron impact (70 eV, source temperature 220 °C).

The volatile components were identified based on the following: (I) comparing their retention times and mass spectra to authentic standards; (II) comparing their mass spectra and retention indices calculated relatively to the C8-C20 n-alkanes on the DB-5 column; (III) comparing their mass spectra in the mass spectra library NIST 2014 (National Technical Information Services, Springfield, VA, USA). The amounts of individual volatile compounds were quantified relative to the internal standard.

### 2.5. Data Analysis

For Y-tube olfactometer assays, the null hypothesis was that pest and natural enemy both showed no preference for each arm (i.e., 50:50 response). The results were analyzed by using a *χ*^2^ goodness-of-fit test, and the unresponsive individuals were not included. Statistical comparisons of volatiles collected from different treatments were carried out by ANOVA followed by the Tukey’s HSD test using the software SPSS 25.0 (IBM SPSS, Somers, NY, USA), and a significance level of *p* < 0.05 was applied. The partial least-squares discriminate analysis (PLS-DA) which defined latent variables as linear combinations of the original manifest variables and calculated to maximize the covariance with the response variables was processed by the software SIMCA-P 14.0 (Umetrics AB, Umeå, Sweden) to test the differences in the cotton plants volatiles under different pest densities. Variable importance for the projection (VIP) value of each compound was also calculated by the software SIMCA-P 14.0, and compounds with a value greater than 1 were considered as contributing separation between each treatment [29].

## 3. Results

### 3.1. Behavioral Responses of Mirids and Parasitoids to Cotton Plants Volatiles

**Figure 1:**
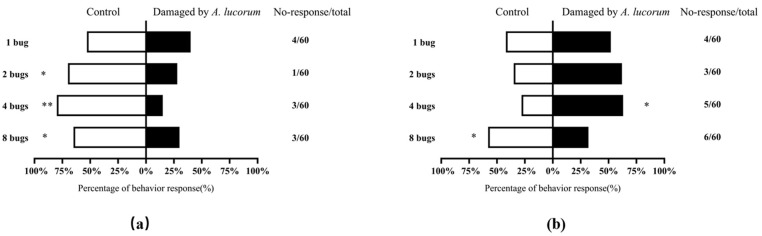
Behavioral responses of insects to damaged cotton plants or health cotton plants volatiles in the Y−tube olfactometer assays. (**a**) behavior responses of *A. lucorum*; (**b**) behavior responses of *P. spretus*. The asterisks were based on the *χ*^2^ analysis. “*” denoted difference at the *p* < 0.05, and “**” denoted difference at the *p* < 0.01.

In dual-choice bioassays, there was no significant difference in adult mirid’s preference between healthy cotton plants and the cotton plants damaged by one mirid bug (*χ*^2^ = 0.594, *df* = 1, *p* = 0.441). However, the adult mirid exhibited a significant response to healthy cotton plants as compared with the cotton plants fed by two or eight mirid bugs (*χ*^2^ = 5.588, *df* = 1, *p* = 0.018; *χ*^2^ = 4.1, *df* = 1, *p* = 0.043). In addition, the mirid adults showed a highly significant response to healthy cotton plants when compared with those damaged by four mirid bugs (*χ*^2^ = 15.395, *df* = 1, *p* < 0.01) (Figure 1a).

The volatiles emitted from cotton plants damaged by four mirid bugs were more attractive to the *P. spretus* adult than those from uninfested cotton plants (*χ*^2^ = 4.328, *df* = 1, *p* = 0.037) while there was no significant difference in the volatiles emitted from cotton plants damaged by one mirid bug or two mirid bugs compared with those from undamaged treatment (*χ*^2^ = 0.333, *df* = 1, *p* = 0.564; *χ*^2^ = 2.286, *df* = 1, *p* = 0.131). It was worth noting that the adult wasp showed a significant response to healthy cotton plants as compared with the cotton plants fed by eight mirid bugs (*χ*^2^ = 4.022, *df* = 1, *p* = 0.045) (Figure 1b).

### 3.2. Volatile Cotton Emission Induced by Different Densities of A. lucorum

**Figure 2:**
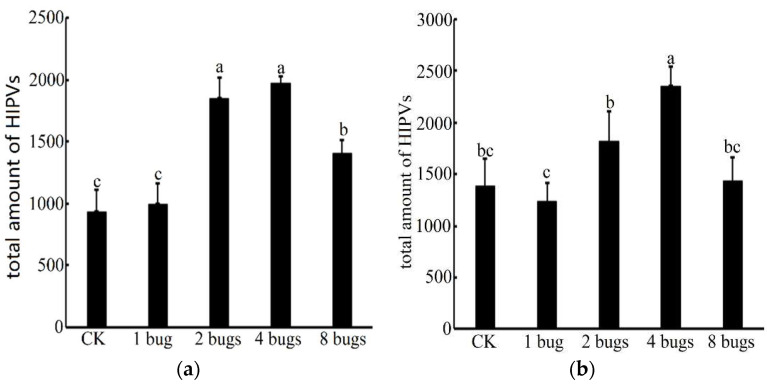
Total amount of HIPVs released from cotton plants and showed by Mean ± SE. Amounts with different letters were significantly different at the 5% level by Tukey’s HSD test. (**a**) Released at the period from 17:00 p.m. to 21:00 p.m.; (**b**) Released at the period from 10:00 a.m. to 14:00 p.m.

From 17:00 p.m. to 21:00, p.m. only 20 volatile compounds were detected from undamaged cotton plants, whereas 27 volatile compounds were detected from the cotton plants damaged by one mirid bug. The number of compounds increased to 31 when the cotton plants were damaged by 4 or 8 mirid bugs, while 30 compounds were found in volatile from cotton plants damaged by two mirid bugs (Table S1). The amounts of HIPVs released at the peak when the cotton plants were damaged by two or four mirid bugs and their total amount were both significantly higher than the rest treatments. The amount from the cotton damaged by eight bugs was also significantly higher than that from undamaged cotton plants or that from cotton plants damaged by 1 mirid bug, while there was no significant difference between undamaged cotton plants and cotton plants damaged by one mirid bug (*F* = 43.862, *p* < 0.05) (Figure 2a).

**Figure 3:**
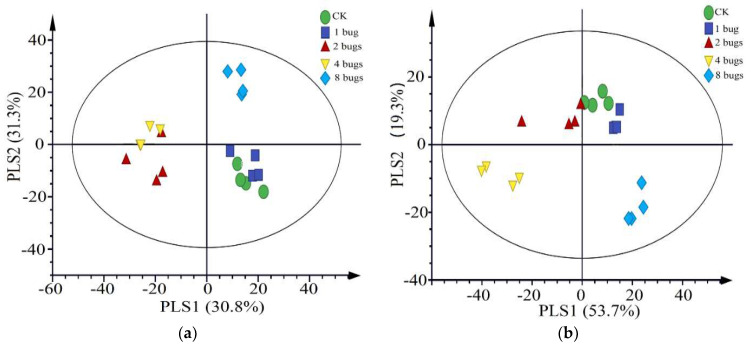
Partial least squares discriminant analysis (PLS-DA) of cotton plants volatile compounds. The cotton plants were either uninfested (Control), infested with one miridae (one bug), two miridae (two bugs), four miridae (four bugs), or eight miridae (eight bugs). The score plot displays the grouping pattern according to the first two components, and the ellipse defines Hotelling’s T2 confidence interval (95%) for the observations. (**a**) HIPVs collected at the period from 17:00 p.m. to 21:00 p.m.; (**b**) HIPVs collected at the period from 10:00 a.m. to 14:00 p.m.

**Figure 4:**
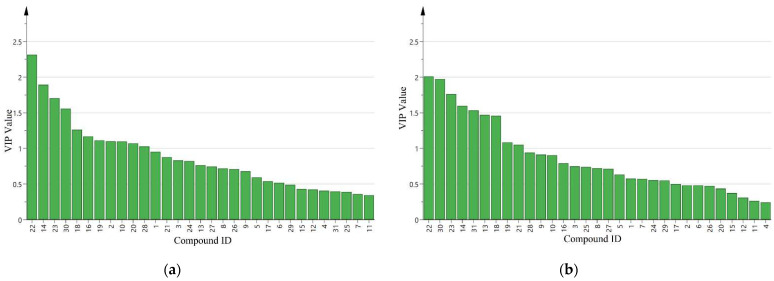
VIP value of cotton plants volatile compounds. (**a**) HIPVs collected at the period from 17:00 p.m. to 21:00 p.m.; (**b**) HIPVs collected at the period from 10:00 a.m. to 14:00 p.m. Compounds ID: 1. ethylbenzene; 2. 1,3-xylene; 3. nonane; 4. *α*-pinene; 5. Benzaldehyde; 6. (*E*)-2-hexenal; 7. *β*-myrcene; 8. 6-methyl-5-hepten-2-one; 9. decane; 10. octanal; 11. terpinene; 12. limonene; 13. (*Z*)-3-hexen-1-yl acetate; 14. (*E*)-*β*-ocimene; 15. acetophenone; 16. linalool oxide; 17. linalool; 18. nonanal; 19. DMNT,(3*E*)-4,8-dimethyl-1,3,7-nonatriene; 20. hexenyl butyrate; 21. (4*Z*)-4-hexenyl butylate; 22. dodecane; 23. decanal; 24. (2*E*)-2-hexen-1-yl propanoate; 25. tridecane; 26. unknown; 27. (*E*)-*β*-caryophyllene; 28. (*E*)-*α*-caryophyllene; 29. *α*-farnesene; 30. (+)-*δ*-cadinene; 31. nerolidol.

After a projection of partial least squares-discriminant analysis (PLS-DA) the contents of detected HIPVs showed a clear separation between uninfested plants and damaged by more than one mirid bug. The first two significant PLS components explained 54.8% and 31.3% of the total variance, respectively (Figure 3a). The first component showed a clear separation between undamaged control cotton plants and cotton plants damaged by two or four mirid bugs, while the second component separated HIPVs released from damaged by eight mirid bugs versus the other treatments. However, the first two components could not separate the healthy plant from the plant damaged by one mirid. The plant damaged by two mirid bugs could not be separated from that damaged by four mirid bugs. In this model, the following volatile compounds dodecane, (*E*)-*β*-ocimene, decanal, (+)-*δ*-cadinene, nonanal, linalool oxide, DMNT, 1,3-xylene, octanal, hexenyl butyrate and (*E*)-*α*-caryophyllene with VIP values ≥ 1.0 contributed most to the separation between the undamaged and *A. lucorum*-damaged volatiles (Figure 4a).

From 10:00 a.m. to 14:00 p.m., a total of 21 volatile compounds were also collected from undamaged cotton plants, whereas 27 were detected from the cotton plants were damaged by one mirid bug. The number of compounds all increased to 31 when the cotton plants damaged by two, four, or eight mirid bugs (Table S2). Analysis of the relative quantities of volatile compounds showed that the total amount of volatiles from the cotton plants damaged by four mirid bugs was significantly higher than other treatments; meanwhile, the amount from cotton plants damaged by two mirid bugs was also significantly higher than the other treatments. However, the number of undamaged cotton plants was not significantly different from the cotton plants damaged by one mirid bug or eight mirid bugs (*F* = 14.285, *p* < 0.05) (Figure 2b).

Partial least squares projection to latent structures-discriminant analysis (PLS-DA) showed a clear separation between undamaged plants and plants damaged by more than one mirid bugs (Figure 3b). The first two significant PLS components explained 53.4% and 20.4% of the total variance, respectively. At this time, the dodecane, (*E*)-*β*-ocimene, decanal, (+)-*δ*-cadinene, nerolidol, (*Z*)-3-hexen-1-ol acetate, nonanal, (*Z*)-butanoic acid, 4-hexenyl ester, and DMNT with VIP values ≥ 1.0 contributed most to the separation (Figure 4b).

## 4. Discussion

When plants perceived pest feeding damage, a series of defensive mechanisms would be activated to promote plant fitness, and therefore herbivores were repelled while the natural enemies were attracted [4,30]. In this study, we investigated the response of *P. spretus* and *A. lucorum* to chemical cues induced by different densities of *A. lucorum*. Our analysis showed that both the *P. spretus* and *A. lucorum* had no obvious preferences for the volatiles from the plant damaged by one bug. Only when the plant was fed by four bugs did the *P. spretus* show an obvious preference, and the *A. lucorum* show an obvious repellency, indicating that defense is activated. This result suggests that there is a positive relationship between the degree of induced resistance in Bt cotton and the density of *A. lucorum* in a certain range.

The pest density was proved to influence the behaviors of herbivorous or carnivorous arthropods in previous studies. For example, after the cotton or lima bean was damaged by *Spodoptera littoralis* Boisd under different densities, *S. littoralis* preferred the plants already damaged by less density or healthy plant [8,31]. A similar conclusion was drawn after testing the behavior of *Plutella xylostella* L. to the cabbage damaged by the same pest under different densities [32]. Besides herbivores, carnivores were also influenced by pest density. *Phytoseius persimilis* Athias-Henriot would not be attracted to kidney beans which were damaged by 30 *Tetranychus urticae* Koch adults until the number reached more than 100 [33]. Similarly, the parasitoid *Cotesia glomerata* L. was only attracted to the *Brussels sprouts* L. damaged by *Pieris brassicae* L. at a high level which indicates that systemic induction does not occur at low-level herbivore infestation [18]. Moreover, when the eggplants were attacked by *Thrips palmi* Karny or *Tetranychus kanzawai* Kishida, the predators *Wollastoniella rotunda* Yasunaga and Miyamoto preferred the odor from the eggplants infested with more pests [34].

The different amounts of HIPVs released in response to the level of infestation were also proved in previous studies [35,36]. In this study, from 10:00 a.m. to 14:00 p.m., the total amount of HIPVs did not significantly increase until the cotton was infested by at least two bugs and the PLS-DA of volatiles revealed a clear separation between cotton damaged by more than one bug versus healthy cotton. From 17:00 p.m. to 21:00 p.m., the total amount of HIPVs significantly increased when the cotton was damaged by four bugs, and the PLS-DA of volatiles only revealed a clear separation between cotton damaged by four or eight bugs versus healthy cotton. This difference might be caused by different rhythm emissions of volatile compounds throughout the day. This phenomenon has been confirmed in other experiments [37]. After an analysis of the chemical components, most of the significantly increased volatile compounds and new compounds were identified as the terpenes, which have been proved to play the most important role in the defense of plants against *A. lucorum* in numerous studies [37,38]. For instance, *β*-myrcene, limonene, and (*E*)-*β*-ocimene were proved to affect the behavior of *A. lucorum* and *P. spretus*; the (+)-*δ*-cadinene was catalyzed to the gossypol, a kind of specialized secondary metabolite that confers resistance to *A. lucorum* when cotton plants perceived damage [39]. Similar to the behavioral changes, most terpenes were only detected from cotton plants damaged by more than one bug and reached a peak when cotton plants were damaged by four bugs. These results imply that an adequately strong elicitation signal which exceeds the damage level threshold was necessary for the inducible resistance. There was a perception that whether plants metabolically reorganized in response to herbivores by inducing the production of defensive materials and reducing major cell processes involved in growth and photosynthesis was determined by the herbivore’s pressures and the cost of the defense [40,41]. Recently a series of studies proved this hypothesis in *Arabidopis thaliana* L., *Brassica nigra* L., and *Zea mays* L. after a pest attack [30,42,43].

In addition, the *P. spretus* showed an obvious preference for the undamaged cotton plants when the cotton plants were fed by eight bugs. It has been initially assumed that the density of herbivores was too high for the plant, so that after pest damage, the plant was not suitable for larval development anymore. Lots of studies have proved that the mother would select the host, which minimizes neonatal mortality because they are at the most sensitive stage of host quality [44,45]. For example, the *Diaeretiella rapae* (McIntosh) only preferred aphid-induced to non-induced plant volatiles at low aphid infestation level, whereas they did not discriminate between volatiles at high aphid infestation level [43]; *Anagrus nilaparvatae* (Pang et Wang) were also only attracted by plant damaged by less than 10 brown planthoppers [46]. A further chemical analysis showed that compared to the cotton plants damaged by four bugs the plant infested by eight bugs did not release any new compound. However, the total amount of HIPVs and the proportions of chemicals in the blend were both changed during the entire infestation. This result implies that the behavior of *P. spretus* might be determined by a blend of multiple compounds and their content ratios in HIPVs, which are considered as a signal from cotton plants to locate hosts. Further investigation on olfactory response and foraging behaviors of *A. lucorum* and *P. spretus* to mixture volatiles should be conducted to test our hypothesis.

In brief, the current study demonstrated that HIPVs emitted by plants in response to *A. lucorum* could be influenced by pest density and could be identified by *P. spretus* as a signal of the host. Our results would help to deeply understand how *P. spretus* plays a role in biological control against *A. lucorum.*

## Data Availability

The data presented in this study are available in Supplementary materials.

## Supplementary Materials

The following are available online at https://www.mdpi.com/article/10.3390/insects12100870/s1, Table S1: Volatile compounds released by differently infested cotton plants collected from 17:00 p.m. to 21:00 p.m.; Table S2: Volatile compounds released by differently infested cotton plants collected from 10:00 p.m. to 14:00 p.m.
