# Supplementary material for: The Effect of Mirid Density on Volatile-Mediated Foraging Behaviour of Apolygus lucorum and Peristenus spretus
Source: Insects. 2021 Sep 25;12(10):870. doi: 10.3390/insects12100870 (PMC8538341; doi:10.3390/insects12100870)
Supplement: Supplementary file 1 [file insects-12-00870-s001.zip › insects-1348599-supplementary.pdf]

Table S1. Volatile compounds released by differently infested cotton plants colleted at the period from 17:00 PM to 21:00 PM. Volatile compounds collected from the headspace of control cotton plants (CK), plants infested with 1 miridae (1 bug), plants infested with 2 miridae (2 bugs), plants infested with 4 miridae (4 bugs), plants infested with 8 miridae (8 bugs). The values represent the mean percentages ± SE of the peak area relative to the peak area of the internal standard. Letters following each value in the same row indicate significant differences between rice plant treatments based on one-way ANOVA followed Tukey HSD test (P<0.05).

Peak no.	Volatile	CK	1 bug	2 bugs	4 bugs	8bugs	p
1	ethylbenzene [1]	30.56±3.24b	32.89±6.19b	29.45±6.68b	29.57±3.12b	63.95±6.20a	P<0.05
2	1,3-xylene [1]	16.49±8.34ab	24.26±7.73a	22.60±3.22a	9.35±0.91b	14.34±0.85ab	P<0.05
3	n-nonane [1]	67.56±9.66c	69.36±0.92bc	74.98±1.96bc	78.96±1.20b	102.7±5.11a	P<0.05
4	*α*-pinene [2]	3.27±1.36a	4.87±1.41a	4.00±0.74a	3.82±0.38a	4.60±0.26a	P=0.194
5	benzaldehyde [2]	14.45±4.71b	10.66±1.93b	31.38±5.01a	28.95±3.10a	13.15±1.98b	P<0.05
6	(*E*)-2-hexenal [2]	15.88±1.66c	15.32±0.56c	27.96±1.51a	21.76±2.26b	6.73±1.15d	P<0.05
7	*β*-myrcene [2]	-	-	8.32±3.03	7.18±1.72	1.13±0.58	
8	6-methyl-5-hepten-2-one [2]	-	19.87±5.53	14.86±8.62	11.05±1.78	12.65±1.29	
9	decane [1]	57.76±9.72a	54.45±4.01a	65.55±3.79a	62.19±2.68a	63.18±11.98a	P=0.268
10	octanal [2]	23.98±3.39b	34.16±17.79b	55.04±5.40a	65.25±5.57a	24.74±5.34b	P<0.05
11	terpinene [1]	-	0.85±0.30	2.55±0.98	2.36±0.57	3.52±1.12	
12	limonene [2]	-	2.02±0.95	4.59±0.70	3.76±0.31	2.24±0.90	
13	(*Z*)-3-hexen-1-yl acetate [2]	11.08±3.40b	9.24±2.07b	44.02±24.24a	33.56±2.16a	10.96±1.73b	P<0.05
14	(*E*)-*β*-ocimene [2]	7.99±2.06c	16.62±5.81c	13.16±6.55c	78.08±18.30a	50.64±7.03b	P<0.05
15	acetophenone [2]	12.32±4.62a	13.32±0.55a	10.36±0.36a	11.46±1.03a	8.5±4.58a	P=0.231
16	linalool oxide [2]	-	11.40±2.97	12.99±2.66	12.21±0.95	10.95±5.18	
17	linalool [1]	19.91±0.75a	17.11±0.81a	20.57±2.05a	19.60±2.31a	17.72±1.53a	P=0.236
18	nonanal [1]	91.08±20.38b	87.85±24.74b	207.69±28.14a	209.92±11.89a	79.45±13.81b	P<0.05
19	DMNT [2,a]	18.59±5.97c	24.41±4.89bc	21.42±5.23c	40.28±2.00a	30.63±3.30b	P<0.05
20	hexenyl butyrate [2]	-	6.75±1.79	3.52±1.29	3.82±1.78	3.66±0.66	
21	(4*Z*)-4-hexenyl butylate [3]	-	11.29±1.20	79.22±11.14	72.76±9.38	12.34±3.02	
22	dodecane [1]	355.28±75.04d	408.65±82.49cd	542.55±130.75bc	623.25±26.62b	763.16±82.99a	P<0.05
23	decanal [1]	108.14±29.48b	60.55±32.69bc	219.93±41.90a	235.25±43.93a	38.30±14.75c	P<0.05
24	(2*E*)-2-hexen-1-yl propanoate [3]	7.01±2.09b	13.60±3.24a	11.99±1.18ab	11.88±1.22ab	15.61±4.08a	P<0.05

25	tridecane [1]	14.97±4.43a	15.44±0.66a	20.38±2.43a	18.30±1.70a	16.28±2.34a	P=0.056
26	unknown	-	-	-	10.70±1.18	10.29±4.25	
27	(*E*)-*β*-caryophyllene [2]	26.64±9.32bc	20.95±0.21c	36.25±4.73a	30.73±2.10ab	1.25±0.39d	P<0.05
28	(*E*)- *α*-caryophyllene [2]	15.78±5.09c	9.37±5.06c	40.75±8.91a	29.78±1.04b	8.65±3.17c	P<0.05
29	*α*-farnesene [2]	-	-	20.59±3.91	19.35±0.99	4.15±1.12	
30	(+)-*δ*-cadinene [2]	-	-	192.24±49.07	174.39±40.12	6.13±2.90	
31	nerolidol [2]	-	1.24±1.09	12.17±2.20	10.87±1.22	2.95±2.56	

Table S2. Volatile compounds released by differently infested cotton plants colleted at the period from 10:00 PM to 14:00 PM. Volatile compounds collected from the headspace of control cotton plants (CK), plants infested with 1 miridae (1 bug), plants infested with 2 miridae (2 bugs), plants infested with 4 miridae (4 bugs), plants infested with 8 miridae (8 bugs). The values represent the mean percentages ± SE of the peak area relative to the peak area of the internal standard. Letters following each value in the same row indicate significant differences between rice plant treatments based on one-way ANOVA followed Tukey HSD test (P<0.05).

Peak no.	Volatile	CK	1 bug	2 bugs	4 bugs	8bugs	p
1	ethylbenzene [1]	14.89±2.33c	18.73±3.49bc	15.97±4.00c	24.11±2.87b	31.24±2.21a	P<0.05
2	1,3-xylene [1]	7.41±2.49b	7.09±0.68b	7.06±1.75b	10.06±1.55b	15.25±5.05a	P<0.05
3	n-nonane [1]	67.72±3.55d	72.25±2.22cd	76.41±3.64c	87.14±6.02b	94.95±4.43a	P<0.05
4	*α*-pinene [2]	3.38±1.10a	4.15±0.70a	3.80±0.66a	5.74±1.39a	5.38±2.56a	P=0.151
5	benzaldehyde [2]	16.26±2.39b	19.73±4.54b	16.92±1.64b	37.56±4.39a	17.63±7.42b	P<0.05
6	(*E*)-2-hexenal [2]	16.36±1.14b	8.36±3.22c	17.68±4.49b	24.79±2.38a	6.43±2.49c	P<0.05
7	*β*-myrcene [2]	-	4.94±1.17	4.83±1.32	6.86±1.05	2.97±0.72	
8	6-methyl-5-hepten-2-one [2]	18.07±7.74a	23.06±4.75a	16.20±8.21a	26.75±7.24a	25.00±2.95a	P=0.157
9	decane [1]	61.47±6.00b	55.57±10.92b	54.20±3.74b	67.59±4.72b	83.04±5.81a	P<0.05
10	octanal [2]	42.95±11.76b	51.20±19.61b	57.19±19.41b	97.05±4.91a	41.28±22.80b	P<0.05
11	terpinene [1]	-	-	2.74±0.64	4.04±0.28	1.74±0.70	
12	limonene [2]	-	0.93±0.55	3.46±0.83	4.97±0.45	2.18±0.48	
13	(*Z*)-3-hexen-1-yl acetate [2]	29.68±13.39bc	15.77±3.89c	44.12±10.80b	87.86±3.18a	100.36±7.72a	P<0.05

14	(*E*)-*β*-ocimene [2]	11.80±2.90b	30.51±17.83b	14.95±7.23b	93.47±10.52a	124.38±48.90a	P<0.05
15	acetophenone [2]	11.15±1.52a	7.94±1.25a	11.31±0.87a	11.07±0.65a	9.47±2.65a	P=0.052
16	linalool oxide [2]	-	9.63±1.47	12.10±3.16	15.31±1.66	8.68±3.02	
17	linalool [1]	21.73±2.25ab	16.45±1.41b	19.27±3.16ab	22.84±4.83a	6.10±2.25c	P<0.05
18	nonanal [1]	157.81±30.19bc	100.91±27.15cd	194.23±48.92b	320.02±23.65a	74.05±36.42d	P<0.05
19	DMNT [2,a]	29.17±10.29b	29.65±4.47b	18.43±4.29c	58.14±2.66a	45.81±17.75ab	P<0.05
20	hexenyl butyrate [2]	-	2.66±0.86	3.61±1.57	5.75±1.47	6.50±4.46	
21	(4*Z*)-4-hexenyl butylate [3]	67.90±16.74b	65.76±10.49b	79.80±25.01b	118.52±24.55a	21.10±11.00c	P<0.05
22	dodecane [1]	530.14±117.92a	471.67±108.98a	602.80±151.50a	427.29±15.75a	552.72±63.74a	P=0.185
23	decanal [1]	232.76±67.27b	250.05±8.3b	249.40±99.78b	386.49±94.04a	32.10±18.28c	
24	(2*E*)-2-hexen-1-yl propanoate [3]	8.16±1.84b	9.57±2.23b	14.11±5.00ab	16.74±2.54a	9.35±2.83b	P<0.05
25	tridecane [1]	11.38±3.44b	13.22±3.12b	13.16±1.76b	13.27±1.13b	36.56±11.34a	P<0.05
26	unknown	-	-	3.31±0.94	6.94±2.00	9.82±2.83	
27	(*E*)-*β*-caryophyllene [2]	19.84±7.60c	23.38±2.15bc	31.05±5.74b	43.00±1.07a	1.22±0.74d	P<0.05
28	(*E*)- *α*-caryophyllene [2]	-	9.40±5.64	33.61±5.53	44.66±1.96	6.70±2.65	
29	*α*-farnesene [2]	-	-	14.82±4.32	26.98±2.02	3.68±1.89	
30	(+)-δ-cadinene [2]	-	-	175.75±37.18	242.55±15.78	20.58±6.78	
31	nerolidol [2]	-	4.49±0.64	13.97±1.26	14.82±4.19	37.06±13.32	

"1" comparing their retention times and mass spectra to authentic standards;

"2" comparing their mass spectra and retention indices calculated relatively to the C8-C20 n-alkanes on the DB-5 column;

"3" comparing their mass spectra in the mass spectra library NIST 2014;

"a" DMNT = (3E)-4,8-dimethyl-1,3,7-nonatriene;

"-" indicates that the concentration of the volatile was below the detection level;

"unknown" were the compounds that were not confirmed with authentic standards.
